# Cannabis-derived cellulose acetate electrospun membranes for therapeutic dressings: extraction, characterization, and prototype development

**DOI:** 10.3389/fchem.2025.1624736

**Published:** 2025-07-08

**Authors:** Adriana Lara, Erika Cely, Edwin Gómez-Pachón, Andres Rubiano-Navarrete, Alex López, Adrian Krzysztof Antosik, Xavier Vendrell, Jarosław Serafin

**Affiliations:** ^1^ Grupo de investigación en diseño, Innovación y Asistencia Técnica para Materiales Avanzados-DITMAV, Escuela de Diseño Industrial, Universidad Pedagógica y Tecnológica de Colombia-UPTC, Duitama, Colombia; ^2^ Grupo de Investigación en Bioeconomía y Sostenibilidad Agroalimentaria, Escuela de Administración de Empresas Agropecuarias, Facultad Seccional Duitama, Universidad Pedagógica y Tecnológica de Colombia, Duitama, Colombia; ^3^ Department of Organic Chemical Technology and Polymer Materials, Faculty of Chemical Technology and Engineering, West Pomeranian University of Technology in Szczecin, Szczecin, Poland; ^4^ Department of Inorganic and Organic Chemistry, University of Barcelona, Barcelona, Spain

**Keywords:** cellulose acetate, electrospinning, nanofibrous membranes, *Cannabis sativa* biomass, wound dressing, adhesive characterization

## Abstract

This work reports the development of electrospun cellulose acetate (CA) membranes derived from *Cannabis sativa* biomass for potential use in therapeutic dressings. Cellulose was extracted from cannabis stalks using alkaline pulping and bleaching, followed by homogeneous acetylation to obtain CA with controlled substitution. CA solutions (13%–25%) were electrospun under varying parameters, and the 17% formulation yielded the most homogeneous, bead-free nanofibers. The resulting membranes were characterized using FTIR, XRD, Raman spectroscopy, UV–Vis spectrophotometry, and SEM. FTIR and Raman confirmed acetylation through characteristic ester and methyl group vibrations. XRD revealed reduced crystallinity in CA compared to native cellulose. SEM analysis showed uniform fiber networks with diameters between 500 and 800 nm. A bilayer dressing prototype was fabricated by integrating the electrospun membrane with a medical-grade silicone adhesive. Adhesion performance was evaluated on synthetic skin using a FINAT-standardized 180° peel test. The membranes demonstrated adequate mechanical cohesion and conformability, supporting their application as sustainable, plant-based biomedical patches.

## 1 Introduction

The rising demand for sustainable, biocompatible materials in biomedical applications is accelerating the transition toward renewable, plant-based alternatives to petroleum-derived polymers (Upadhyay et al., 2023). Among natural polymers, cellulose stands out due to its biodegradability, high tensile strength, and ability to undergo chemical modification (Erdal and Hakkarainen, 2022). Another significant advantage of cellulose is its natural abundance and widespread availability in lignocellulosic biomass, making it a cost-effective and renewable platform for material design. Its acetylated derivative, cellulose acetate (CA), has found widespread application in membranes, biomedical, drug delivery systems, and wound dressings, thanks to its biocompatibility, film-forming properties, and structural versatility (Seddiqi et al., 2021; Fatema et al., 2022; Bifari et al., 2016). Conventionally, CA is synthesized from wood pulp or cotton linters; however, these sources are associated with ecological drawbacks and increasing economic pressure, motivating the exploration of lignocellulosic residues as alternative feedstocks (Goh et al., 2021). *Cannabis sativa* stalks, particularly the bast fiber fraction, present a chemically favorable and socioeconomically strategic alternative for cellulose extraction. Structurally, bast fibers contain up to 70% α-cellulose, characterized by high crystallinity and low lignin content (typically <10%), which reduces the need for aggressive delignification and facilitates cleaner downstream processing. This makes them especially suitable for homogeneous acetylation reactions, where purity and chain accessibility significantly influence substitution efficiency and final polymer performance. Moreover, the high hemicellulose-to-lignin ratio and reduced ash content further distinguish cannabis bast fibers from wood pulp or cotton linters, both of which require extensive pretreatment or bleaching. From a sustainability perspective, *Cannabis sativa* is a fast-growing, low-input crop requiring significantly less water, land, and pesticide input than cotton or forest biomass, enabling a reduced environmental footprint throughout the agricultural phase. Importantly, in several countries, cannabis biomass particularly from unregulated cultivation is produced in large quantities but lacks formalized industrial applications, leading to accumulation of agricultural waste. In Latin America and parts of Eastern Europe, for instance, cannabis residues are often burned or left unused, despite their biochemical value. Leveraging this biomass aligns with circular economy objectives by valorizing a problematic, underutilized feedstock into advanced medical products. Furthermore, promoting cannabis-based biopolymers in regulated biomedical sectors can catalyze socioeconomic change by transforming informal agricultural practices into legally recognized, innovation-driven value chains. In this context, *Cannabis sativa* offers not only a technically advantageous source of high-purity cellulose, but also a politically and economically meaningful path toward green development. In parallel, industrial hemp (*Cannabis sativa*) has gained renewed interest not only as a sustainable crop but also as a promising source of cellulose (Visković et al., 2023). Bast fibers from hemp stalks can contain up to 70% cellulose and require significantly fewer agrochemical inputs compared to cotton or timber sources. Despite this, the fibers are often underutilized or discarded, even in regions where cannabis cultivation is widespread. In countries facing socioeconomic instability, unregulated cannabis production is frequently linked to informal economies and organized crime (Crini et al., 2020). Valorizing cannabis biomass into high-value biomedical materials presents an opportunity to replace informal supply chains with legal, technology-based industries that contribute to regional development, sustainability, and public health innovation (Frusciante et al., 2025). While hemp fibers have been used in textiles, construction, and composites, their conversion into biomedical-grade polymers remains rare (Zimniewska, 2022). To date, no studies have reported the synthesis of cellulose acetate from cannabis-derived cellulose for use in electrospun wound dressings, representing a significant gap in both sustainable material development and bioeconomic potential. This is particularly relevant given the global wound care market valued at over USD 23 billion in 2024 which continues to grow due to aging populations, increasing rates of chronic wounds, and rising demand for high-performance, biodegradable dressings (Grand view research, 2025). Electrospinning is a powerful technique for producing nanofibrous membranes with high porosity, surface area, and tunable fiber diameter characteristics that closely mimic the extracellular matrix and support wound healing processes (Gao et al., 2021). Electrospun CA membranes derived from commercial sources have shown favorable results in promoting epithelial regeneration, antimicrobial delivery, and tissue repair (Chen et al., 2024). However, the electrospinnability and membrane performance of CA synthesized from non-conventional sources such as *Cannabis sativa* remain completely unexplored (Spizzirri et al., 2021). The properties of CA especially solubility, viscosity, and fiber-forming behavior depend critically on the cellulose source and the acetylation method (Han et al., 2022). Homogeneous acetylation, using glacial acetic acid and controlled catalysis, offers better control over degree of substitution and molecular uniformity, which are essential for achieving stable, bead-free electrospinning (Teixeira et al., 2020). Despite advances in nanofiber fabrication, a recurring limitation in literature is the lack of functional device translation. Many studies stop at structural characterization of electrospun membranes, neglecting critical performance attributes such as mechanical cohesion, skin adhesion, and clinical usability (Olteanu et al., 2024). This is a crucial shortcoming, as wound dressings must adhere effectively, adapt to skin movement, and maintain integrity under moisture and mechanical stress (Farahani and Shafiee, 2021). Integration with pressure-sensitive adhesives and evaluation via standardized peel testing such as the FINAT 180° method—are essential steps in validating membrane performance for real-world biomedical applications (Georgiou et al., 2025). Yet very few natural polymer systems have been taken this far in development, especially those derived from novel, sustainable biomass (Mohanty et al., 2022).

This study directly addresses these gaps by presenting an integrated approach to the development of nanofibrous wound dressing membranes from cellulose acetate synthesized from *Cannabis sativa* bast fibers. It aims to establish a sustainable and clinically relevant workflow that spans from cellulose extraction and homogeneous acetylation to electrospinning of CA membranes and integration into a bilayer dressing system with silicone-based pressure-sensitive adhesion. In contrast to conventional studies, this work does not stop at material fabrication or surface analysis; it extends to functional validation under clinically relevant mechanical testing conditions, including peel strength, tack, cohesion, and shrinkage tests aligned with FINAT standards. To the best of our knowledge, this is the first study to utilize *Cannabis sativa*-derived cellulose to synthesize electrospinnable cellulose acetate, to fabricate uniform nanofibrous membranes from this unconventional source, and to integrate them into adhesive wound dressing prototypes evaluated through standardized performance testing. The results demonstrate a sustainable, scalable pathway to convert agricultural residues into high-performance biomedical materials, with direct implications for therapeutic innovation, waste valorization, and regional economic development within the framework of a circular bioeconomy.

## 2 Materials

Residual *Cannabis sativa* stalks were collected in Sotaquirá, Boyacá, Colombia, and used as the cellulose source. Sodium hydroxide (NaOH, analytical grade), hydrogen peroxide (H_2_O_2_, 30%), and glacial acetic acid (CH_3_COOH, ≥99.7%) were purchased from Química Latina S.A.S. and Quimcol S.A. (Bogotá, Colombia). Acetic anhydride (≥98%) and sulfuric acid (H_2_SO_4_, 98%) were obtained from Sigma-Aldrich (United States) and J.T. Baker (United States), respectively, and used for homogeneous acetylation. Distilled water was used throughout all purification and neutralization steps. Commercial cellulose acetate (Mn ∼30,000, DS ∼2.5, Sigma-Aldrich, United States) was used for electrospinning calibration. DOWSIL™ 7388 (Q2-7388) silicone adhesive was supplied by Dow Inc. (United States); it is a clear, high-temperature-resistant resin (viscosity: 20,000–80,000 mPa·s; nonvolatile content: 55%–58%). Dichlorobenzoyl peroxide (DClBPO >98%), used as a thermal crosslinking initiator, was obtained from Novichem (Poland).

## 3 Methodology

### 3.1 Extraction of cellulose pulp

Residual biomass of *Cannabis sativa*, consisting of stems and branches, was collected from the municipality of Sotaquirá, Boyacá, Colombia (approximate elevation: 2,860 m above sea level; ambient temperature range: 6°C–19°C). The biomass was first air-dried at room temperature to remove moisture, then manually cleaned to eliminate visible impurities such as soil. Stem and branch fractions were separated to ensure material consistency. The dried biomass was mechanically processed in two stages: coarse crushing using a Trapp JTRF70 mill and subsequent fine grinding using a GARDOM CG9435A grinder. In this stage, crushed plant material was milled for 15 s per batch, which facilitated partial separation of woody material from bast fibers and increased surface area for downstream treatments.

Initial pretreatment was conducted via alkaline hydrolysis to remove surface impurities such as waxes, fats, pectins, and colorants. Stem and branch fibers were separately immersed in a 0.35% (w/v) sodium hydroxide (NaOH) solution, at a solid-to-liquid ratio of 1:20 (w/v), and heated to 90°C for 90 min. Afterward, the fibers were thoroughly washed with distilled water to eliminate residual solubilized components and extract pigments. Neutralization was carried out using glacial acetic acid (CH_3_COOH) in a 1:1 (v/v) ratio until pH 7 was reached. The neutralized fibers were oven-dried at 60°C for 12 h. This procedure was adapted from the method reported by Solís García et al. (2025). For delignification and removal of residual non-cellulosic material, the dried fibers underwent a second chemical treatment using 10% (w/v) NaOH at a 1:50 solid-to-liquid ratio and 11% (v/v) hydrogen peroxide (H_2_O_2_) at a 1:25 ratio. The alkaline-oxidative treatment was conducted at boiling temperature (100°C) for 120 min, following the procedure reported by Solís García et al. (2025). The treated fibers were then washed with distilled water and neutralized again using glacial acetic acid (pH adjusted to 7). After drying, visible woody particles remaining in the fiber matrix were manually removed to ensure the purity of the cellulose. The efficiency of the cellulose extraction process from dried *Cannabis sativa* stalks was calculated based on the dry mass of purified cellulose obtained after alkaline–oxidative treatment. The average extraction yield was 32% ± 2% w/w (n = 3). Similarly, the conversion yield of purified cellulose into cellulose acetate through homogeneous acetylation was 87% ± 3% w/w, based on the dry weight of the final polymer. These results indicate high process efficiency for both biomass valorization and functional derivatization.

Homogeneous Acetylation of *Cannabis sativa* Cellulose. The cellulose obtained was subjected to homogeneous acetylation to synthesize cellulose acetate. Following the protocol described by (Homem and Amorim, 2020), 2.0 g of dried cellulose were dispersed in 40 mL of glacial acetic acid and stirred for 30 min to allow complete swelling. A catalytic solution containing 0.2 mL of concentrated sulfuric acid and 17.5 mL of glacial acetic acid was then added, and the mixture was stirred for an additional 15 min. The entire acetylation process was conducted at ambient (room) temperature (17°C–20°C). Subsequently, 20 mL of acetic anhydride was introduced to initiate acetylation, followed by continuous stirring for another 30 min. The reaction mixture was left undisturbed at room temperature for 24 h to complete the substitution reaction. To precipitate the cellulose acetate, 100 mL of distilled water was added slowly under stirring, and the system was stirred for 1 h. The resulting precipitate was collected by vacuum filtration and washed repeatedly with distilled water until a neutral pH was reached. The final product was air-dried and stored under dry conditions for subsequent use. Drying was performed at ambient temperature (17°C–20°C) for 48 h under desiccation conditions.

### 3.2 Electrospinning

Electrospinning experiments were performed using a laboratory-scale electrospinning unit developed in-house at the Universidad Pedagógica y Tecnológica de Colombia. Prior to spinning the synthesized cellulose acetate, a series of tests were conducted using commercial cellulose acetate (Sigma-Aldrich, Mn ∼30,000, DS ∼2.5) to calibrate and optimize the equipment and spinning parameters. Polymer solutions were prepared by dissolving cellulose acetate in a binary solvent mixture of acetone and N,N-dimethylformamide (DMF) at a 2:1 (v/v) ratio. Different polymer concentrations were evaluated to determine the optimal viscosity for uniform fiber formation. Based on previous reports (Ochica et al., 2016), the electrospinning process was carried out using a flow rate of 0.5 mL/h, a needle-to-collector distance of 12 cm, and an applied voltage range of 13–20 kV, adjusted depending on solution properties. All operations were performed under ambient temperature and humidity conditions (Ochica et al., 2016).

### 3.3 Characterization

The chemical structure of the fibers and derived materials was analyzed by Fourier-transform infrared spectroscopy (FTIR) using a Thermo Scientific Nicolet iS5 equipped with an ATR iD7 module. Spectra were acquired over the 400–4,000 cm^−1^ range, the resolution was set to 4 cm^−1^, and spectra were collected over 64 scans per sample, to evaluate the removal of hemicellulose and lignin after alkaline treatment. Crystallinity and phase changes in raw and processed samples including untreated cannabis cellulose, commercial cellulose, synthesized cellulose acetate, and electrospun membranes were determined by X-ray diffraction (XRD) using a PANalytical Aeris diffractometer with Cu Kα radiation (λ = 1.5406 Å), operating at 40 kV and 100 mA, scanning from 15° to 60° 2θ at 0.15°/min. The average crystallite size was calculated using a Scherrer equation (Serafin et al., 2023). The Scherrer equation (Equation 1) is given by:

$$B\left(2\theta \right)=\frac{K\lambda }{Lcos\theta }$$ (1)

Where: Peak width (B) or Full Width at Half Maximum (FWHM) is inversely proportional to crystallite size (L); λ is the wavelength of X-ray used and θ is the glancing angle.

Raman spectroscopy was carried out on a Jobin-Yvon LabRaman HR 800 system (CCiTUB) using a 532 nm laser at 1.5 mW to avoid thermal effects; spectra were obtained under ×50 magnification with three accumulations of 5 s each. UV–Vis spectra (200–800 nm) were recorded in reflectance mode using a Shimadzu UV-3600 spectrophotometer to characterize solid samples of both synthesized cellulose acetate and electrospun membranes. Surface morphology and fiber uniformity of the membranes were examined by scanning electron microscopy (SEM) using a JSM-7100F thermal field emission microscope; gold-coated samples were analyzed to assess fiber continuity, average diameter, and surface defects.

### 3.4 Obtaining the prototype of therapeutic dressing

A silicone-based transfer-type self-adhesive film was prepared by mixing DOWSIL™ 7388 silicone pressure-sensitive adhesive with 2–4 wt% of dichlorobenzoyl peroxide (DClBPO) as a thermal crosslinking initiator. The mixture was homogenized and coated at a density of 45 g/m^2^ onto a fluorosilicone-coated polyester release liner (50 g/m^2^) using a semi-automatic film applicator. Crosslinking was performed by thermal curing at 110°C for 10 min. The adhesive laminate was then covered with a second fluorosilicone-coated polyester film and cut into standardized strips for assembly and mechanical testing. For dressing assembly, the adhesive film was transferred onto a fabric-based protective backing layer. The electrospun cellulose acetate membrane was then centrally laminated onto the adhesive region, leaving free edges of exposed adhesive to ensure proper fixation to the skin. The assembled dressing was sealed with an additional protective polyester liner to maintain structural integrity prior to evaluation.

The adhesive performance of the dressing system was assessed according to FINAT international standards. Peel adhesion was measured using FINAT FTM 1 (180° angle, 300 mm/min) at 70°C. Cohesion (shear resistance) was tested in accordance with FINAT FTM 8 at both room and elevated temperatures. Tack was evaluated by loop testing following FINAT FTM 9, while dimensional stability (shrinkage) was analyzed using FINAT FTM 14 (Anderson et al., 2010; Kamperman et al., 2010; Kowalski et al., 2013; Antosik et al., 2020; Bartkowiak et al., 2020; Antosik and Mozelewska, 2022).

## 4 Results

**FIGURE 1:**
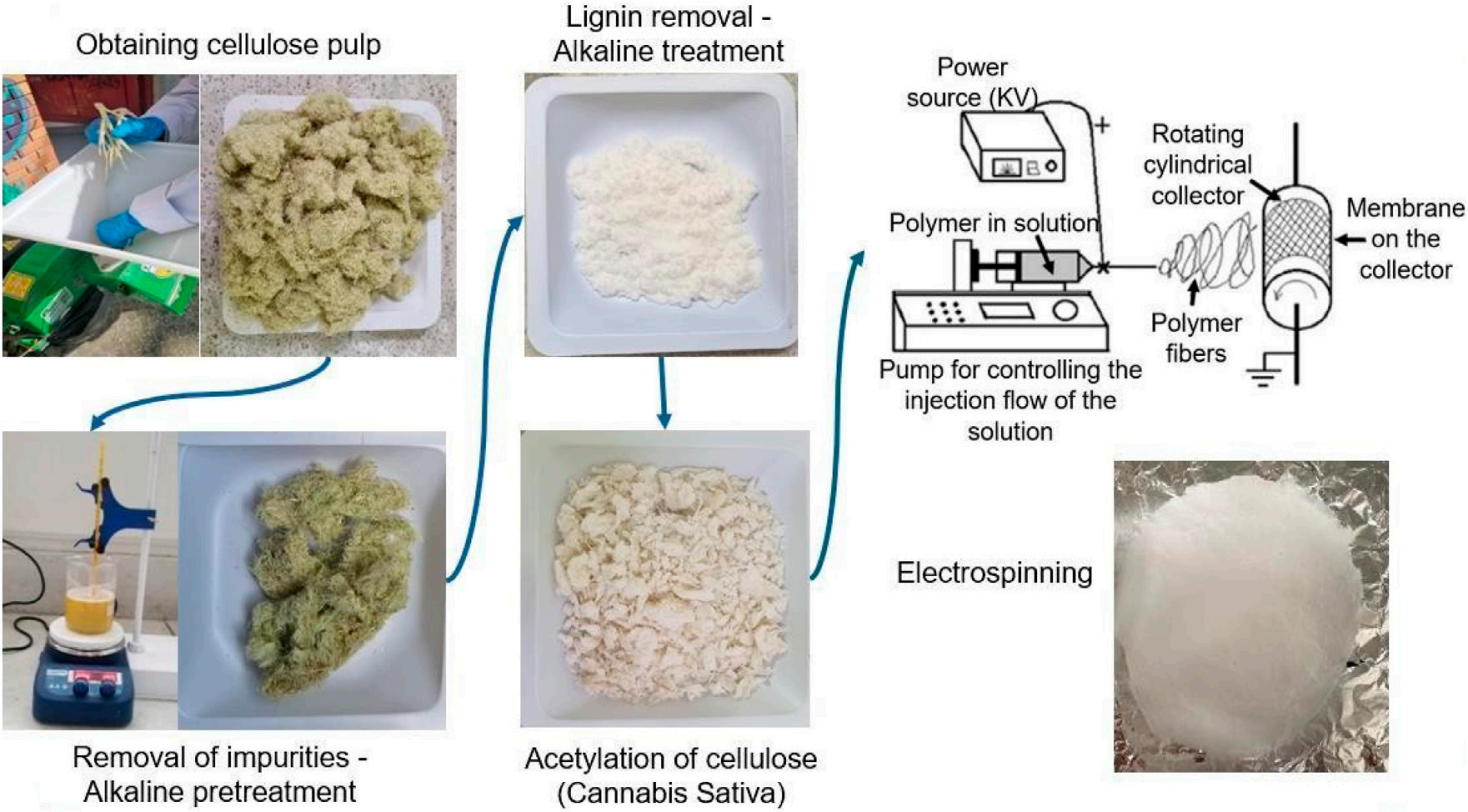
Process results.

The recovery of cellulose from *Cannabis sativa* stalks began with mechanical size reduction to facilitate separation of woody residues and enhance surface area for subsequent chemical processing. The biomass was initially crushed using a Trapp JTRF70 mill and then finely ground with a GARDOM CG-9435A unit. Alkaline hydrolysis effectively removed surface impurities such as waxes, fats, pectins, and pigments. A subsequent alkaline–oxidative treatment enabled efficient delignification and bleaching, yielding a purified cellulose pulp. The cellulose was acetylated via homogeneous synthesis under acid-rich conditions, producing a gel-like cellulose acetate with enhanced viscosity and solubility in organic solvents. To optimize electrospinning, a series of tests were conducted using commercial cellulose acetate (Sigma-Aldrich) as a reference material. Polymer solutions at concentrations of 13%, 15%, 17%, 19%, and 25% (w/v) were prepared in a 2:1 acetone:N,N-dimethylformamide (DMF) system (Table 1). Among these, the 17% solution demonstrated optimal viscosity and fiber-forming stability, whereas other concentrations exhibited either low consistency or excessive atomization. Based on these trials, electrospinning was performed using the following optimized parameters: 17% polymer concentration, 0.5 mL/h flow rate, 17 kV voltage, 12 cm needle-to-collector distance, and 5 mL injection volume. The process yielded uniform nanofibrous membranes after approximately 2 h of continuous electrospinning. A schematic overview of the process, including cellulose extraction, acetylation, and membrane fabrication, is shown in Figure 1.

**TABLE 1:** Experimental design, variation of electrospinning parameters.

Parameter	Experiment 1	Experiment 2	Experiment 3	Experiment 4	Experiment 5
Polymer concentration (%)	13	15	17	19	25
Flow rate (mL/h)	0.5	0.5	0.5	0.5	0.5
Applied voltage (Kv)	13	15	16	18	20
Needle-collector distance (cm)	12	12	12	12	12

Source: Author.

Different tests were carried out in Table 1. To standardize the electrospinning process using commercial cellulose acetate from Sigma–Aldrich, polymer concentration and fiber formation response.

### 4.1 Fourier transform infrared spectroscopy (FTIR)

**FIGURE 2:**
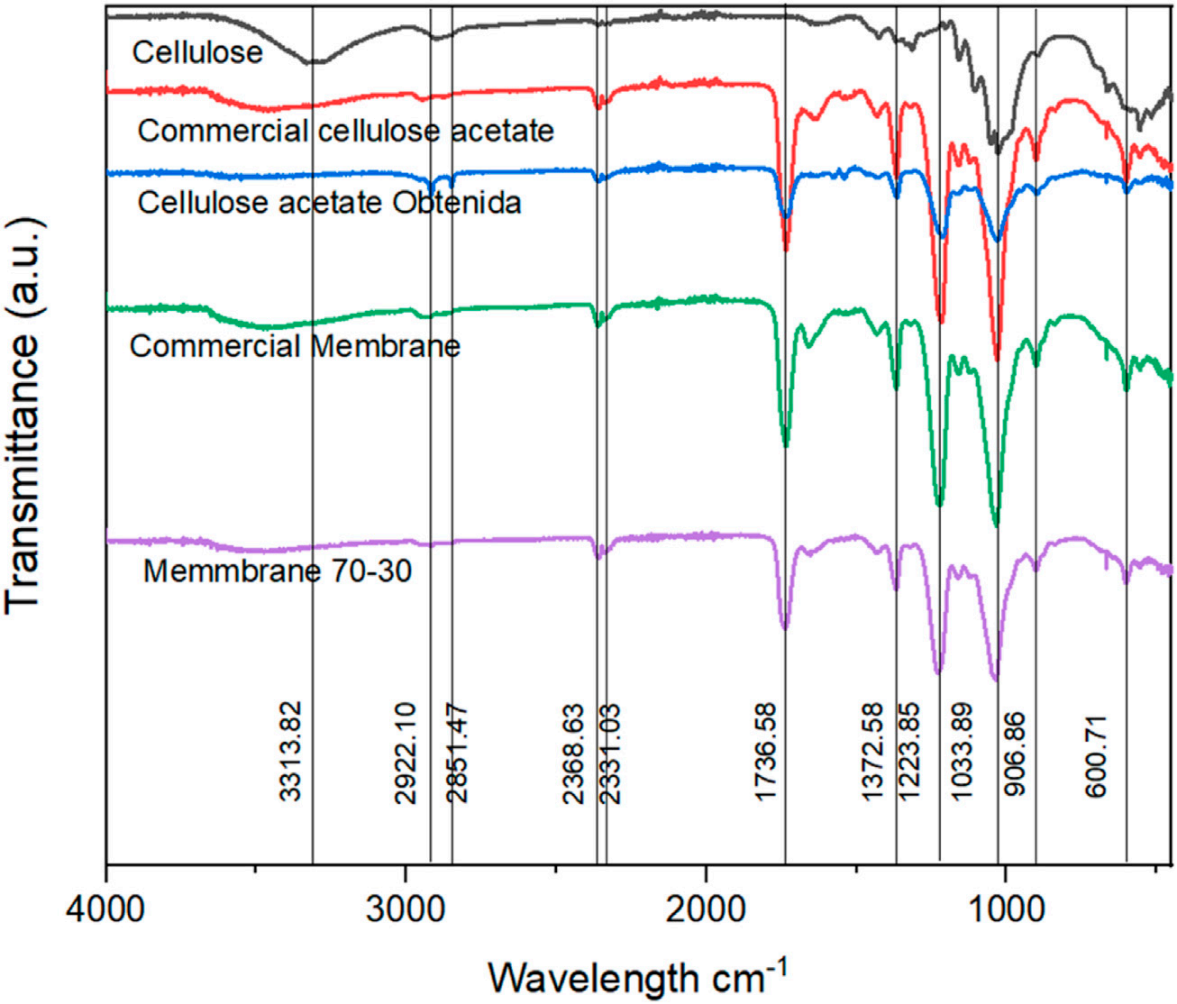
FTIR spectra of extracted cellulose, synthesized cellulose acetate, and electrospun membranes derived from *Cannabis sativa*.

FTIR spectroscopy was used to monitor the chemical structure of native cellulose extracted from *Cannabis sativa* biomass, the synthesized cellulose acetate, and the electrospun membranes derived therefrom. Figure 2 presents the overlaid FTIR spectra for each sample, revealing key vibrational signatures corresponding to the functional groups of cellulose and its esterified derivative. In the native cellulose spectrum, a broad and intense absorption band centered at 3313.82 cm^−1^ is attributed to O–H stretching vibrations. This band reflects the strong intermolecular and intramolecular hydrogen bonding network among the hydroxyl groups on the C2, C3, and C6 positions of the glucopyranose units. The breadth of this band arises from the distribution of hydrogen bond strengths in the semi-crystalline cellulose matrix. Upon acetylation, this band is significantly attenuated, reflecting the substitution of hydroxyl groups with acetyl moieties and thus a reduced hydrogen bonding capacity. C–H stretching vibrations of methyl and methylene groups appear in the 2,900–3,000 cm^−1^ region across all spectra. Specifically, symmetric and asymmetric C–H_2_ stretching modes are observed above 2,990 cm^−1^, confirming the retention of the polysaccharide backbone post-modification (Cichosz and Masek, 2019). These vibrations are less sensitive to chemical substitution but are essential for validating the structural continuity of the cellulose chain. A sharp band at 1736.58 cm^−1^, present only in the cellulose acetate and membrane samples, corresponds to the C=O stretching vibration of ester groups. This absorption is a direct spectral fingerprint of successful acetylation, indicating the formation of O-acetylated glucose residues through the esterification of the hydroxyl functionalities. The precise position and intensity of this band depend on the degree of substitution (DS), solvent environment, and sample crystallinity. To quantify the extent of acetylation, the degree of substitution (DS) was estimated semi-quantitatively based on the FTIR spectra. Following the method outlined by Wolfs et al. (2023), DS was calculated from the ratio of absorbance intensities of the ester carbonyl stretch (∼1736 cm^−1^) to the glycosidic C–O–C stretch (∼1,030 cm^−1^) using the following equation (Equation 2):

$$DS=K\cdot \left(\frac{A_{1736}}{A_{1030}}\right)$$ (2)

where A_1736_ and A_1030_ are the peak absorbances of the C=O and C–O–C bands, respectively, and KKK is a calibration constant typically ranging from 2.8 to 3.0. The calculated DS for the cannabis-derived cellulose acetate was approximately 2.3, indicating a triacetate-like structure. This level of substitution ensures solubility in acetone/DMF mixtures and facilitates bead-free electrospinning, as confirmed by SEM (see Figure 5). The FTIR-derived DS value supports the processability and functional suitability of the synthesized polymer for biomedical nanofiber applications. Its absence in the native cellulose confirms the specificity of the reaction. Another notable feature is the band at 1,372.58 cm^−1^, arising from the symmetric bending vibrations (scissoring) of methyl (–CH_3_) groups introduced during acetylation. This vibration is a secondary indicator of acetyl group incorporation and is typically used in conjunction with the C=O band to confirm structural modification. Together, these two bands are widely used to calculate the DS through semi-quantitative spectral methods. The glycosidic C–O–C stretching vibrations appear prominently in the range of 1223.85 to 1033.89 cm^−1^. These bands are associated with the asymmetric and symmetric stretching of ether linkages between the β-1,4-linked glucose units in the cellulose chain. Their persistence across all samples confirms the preservation of the main polymer backbone after both alkaline purification and acetylation. A minor band observed at 908.86 cm^−1^ is assigned to the out-of-plane bending of C–H bonds, while the band at 600.71 cm^−1^ corresponds to in-plane bending of C–OH, both contributing to the spectral fingerprint region. These modes, although less intense, are sensitive to conformational changes and provide additional insight into local structure. Importantly, the FTIR spectra of the electrospun membranes display no new peaks or significant shifts relative to the cellulose acetate spectrum, indicating that the electrospinning process did not induce degradation, crosslinking, or unexpected side reactions. This chemical stability post-fabrication is essential for biomedical applications, where consistent surface chemistry influences biocompatibility, hydrophilicity, and drug loading capacity. When compared to reference spectra reported by (Wolfs et al., 2023), the extracted cellulose shows excellent correspondence in all major vibrational regions, including O–H, C–H, and C–O–C modes. This confirms the efficacy of the alkaline and oxidative treatments in removing lignin, hemicellulose, and other interfering components, resulting in highly purified cellulose suitable for functional modification. The overall spectral integrity across all samples further validates the synthetic route and confirms the successful transformation of *Cannabis sativa* biomass into a structurally preserved, chemically modified biopolymer.

### 4.2 X-ray diffraction (XRD)

**FIGURE 3:**
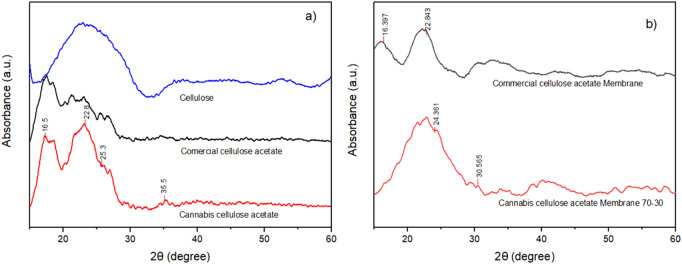
XRD patterns of **(a)** native *Cannabis sativa* cellulose, synthesized cellulose acetate, and commercial cellulose acetate, and **(b)** electrospun membranes produced from cannabis-derived and commercial cellulose acetate.

X-ray diffraction was employed to evaluate the crystalline and amorphous domains of *Cannabis sativa*-derived cellulose, the synthesized cellulose acetate, and their corresponding electrospun membranes. Figure 3 shows the diffractograms for native cellulose and cellulose acetate (Figure 3a), as well as for the electrospun membranes in comparison to commercial analogues (Figure 3b). The XRD profile of extracted *Cannabis sativa* cellulose exhibits three distinct diffraction peaks centered at 2θ ≈ 16.5°, 22.8°, and 35.5°, which correspond to the (110), (002), and (004) crystallographic planes of cellulose I, respectively. These assignments are consistent with the monoclinic crystalline structure of native cellulose (cellulose Iβ), as previously reported by (Gallina et al., 2024). The intense reflection at 22.8°, attributed to the (002) plane, is indicative of the highly ordered packing of glucopyranose chains, supported by inter- and intramolecular hydrogen bonding. The sharpness and intensity of this peak suggest a relatively high crystallinity index (CrI), a key factor in mechanical integrity, thermal resistance, and enzymatic recalcitrance of cellulose-based materials. Compared to commercial cellulose, the diffraction pattern of the cannabis-derived cellulose shows similar peak positions and relative intensities, indicating successful extraction and purification without substantial loss of native crystallinity. This suggests that the alkaline and oxidative treatments were effective in removing amorphous hemicellulose and lignin fractions while preserving the intrinsic ordered regions of cellulose. In contrast, the diffraction pattern of the synthesized cannabis cellulose acetate presents significant morphological changes. The spectrum reveals a broad, low-intensity halo centered around 2θ ≈ 17.5°, accompanied by the loss of sharp crystalline reflections. This broad feature is characteristic of amorphous cellulose acetate, where the introduction of bulky acetyl groups at the C2, C3, and C6 positions of the anhydroglucose units disrupts the original hydrogen bonding and crystalline packing. The acetylation process, particularly under homogeneous conditions, leads to a breakdown of the original cellulose lattice and generates a random coil-like structure with high conformational freedom. Interestingly, a weak reflection around 2θ ≈ 25.3° is observed in the cannabis cellulose acetate spectrum (Park et al., 2023). This may be attributed to residual or reorganized short-range crystalline domains resulting from incomplete substitution or partial molecular alignment during solvent evaporation. Such partial crystallinity, while reduced, may contribute to improved dimensional stability and mechanical resistance in the final material a balance often sought in membrane and film applications. The XRD pattern of the electrospun membrane produced from cannabis-derived cellulose acetate also exhibits a broad amorphous halo near 17°, similar to its cast precursor, indicating that the electrospinning process did not significantly alter the molecular organization. No additional crystalline reflections or structural degradation peaks were observed, confirming that the high-voltage spinning conditions preserved the polymeric structure. The diffuse nature of the membrane pattern supports its suitability for applications where high flexibility, controlled porosity, and permeability are required, such as wound dressings or drug delivery platforms. A comparative analysis with the commercial cellulose acetate membrane (Figure 3b) reveals similar amorphous features, suggesting structural and processing equivalence. Both display characteristic halos in the same angular range (16°–18°), further validating the efficacy of the synthetic and processing routes used for the cannabis-based material. Furthermore, using the Scherrer equation to estimate crystallite size from the main diffraction peaks, the commercial cellulose acetate membrane exhibited a crystallite size of approximately 8.1 nm, while the cannabis-derived cellulose acetate membrane showed a slightly smaller value of 6.8 nm. This reduction may reflect the presence of less-ordered short-range domains or broader molecular weight distribution in the biomass-derived sample, consistent with its more diffuse diffraction profile.

### 4.3 Raman spectroscopy

**FIGURE 4:**
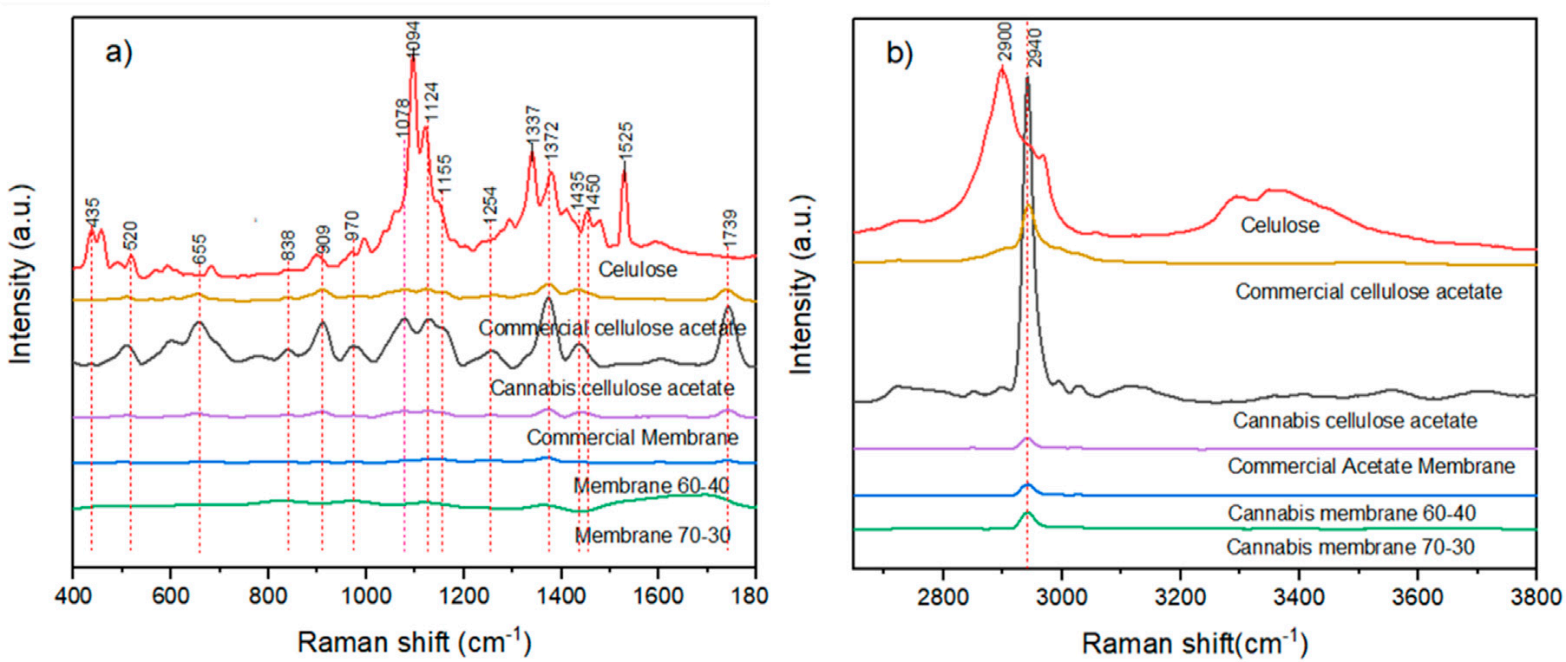
Raman spectra of cellulose, cellulose acetate, and electrospun membranes derived from *Cannabis sativa*: **(a)** 400–1800 cm^−1^ fingerprint region, **(b)** 2,600–3800 cm^−1^ high-wavenumber region.

The Raman spectrum consists of bands caused by inelastic scattering of chemically bonded structures, as shown in Figures 4a,b.

Raman spectroscopy was conducted to probe molecular-level structural transformations during the chemical modification of *Cannabis sativa*-derived cellulose into cellulose acetate and its electrospun membranes (see Figure 4). The collected spectra (Figure 4a, 400–1,800 cm^−1^; Figure 4b, 2,600–3,800 cm^−1^) provide detailed insights into changes in bond environments, hydrogen bonding networks, and chain conformations. These results were analyzed in direct comparison with well-established vibrational assignments in the literature for cellulose I, II, and cellulose acetate polymorphs. In the fingerprint region, the native cellulose spectrum shows vibrational bands at ∼435 cm^−1^ and ∼520 cm^−1^, corresponding to skeletal bending modes of C–C and C–O–C bridges within the glucopyranose ring. These are sensitive to ring puckering and torsion, and their disappearance in acetylated samples reflects the loss of rigidity and increased ring distortion due to acetyl substitution. Between 659 and 978 cm^−1^, the spectrum contains C–O and O–H in-plane bending and deformation vibrations, especially sensitive to hydroxyl presence and hydrogen bonding strength consistent with (Taher et al., 2023). The suppression of these modes in cellulose acetate confirms the substitution of hydroxyl groups at C2, C3, and C6 with acetyl moieties, leading to steric hindrance, reduced hydrogen bonding, and increased segmental mobility. The disappearance of these bands’ mirrors established Raman profiles of cellulose triacetate (Satha et al., 2020). The band at 1,081 cm^−1^ originates from ring-breathing modes and coupled C–O stretching, while 1,094 cm^−1^ includes asymmetric glycosidic C–O–C stretching and CH_2_ wagging. These bands are critical for determining the degree of polymerization and backbone integrity. Their persistence post-acetylation and post-electrospinning confirms that the β-1,4-glycosidic linkages remain chemically intact throughout processing a nontrivial observation given the strong acid catalysis in acetylation. It further supports the Raman selection rule sensitivity to long-range order in polysaccharides. The 1,121 cm^−1^ peak, also due to asymmetric glycosidic stretching, and the 1,265 cm^−1^ band (C–OH deformation) diminish in acetylated samples, further confirming the progressive elimination of hydrogen bond donors and acceptors. These results align closely with the characteristic Raman spectra of semi-substituted cellulose acetate (DS ∼2.3) reported in polymer structural studies (Atykyan et al., 2020). Crucially, acetylation introduces new vibrational features. The peaks at 1,377, 1,382, 1,435, and 1,479 cm^−1^ correspond to CH_3_ symmetric bending, CH asymmetric deformation, and H–C–H scissoring motions, respectively modes Raman-active due to changes in polarizability tensor from asymmetrical substitutions. These vibrations increase in intensity with DS, serving as diagnostic markers of substitution. The appearance of a sharp peak at 1736 cm^−1^, corresponding to the ν(C=O) stretch in esters, unambiguously confirms successful acetylation. Its sharpness reflects homogeneity in substitution pattern and supports the presence of triacetate domains. In the high-frequency region, the aliphatic C–H stretching near 2,934 cm^−1^ is present in all samples, reflecting CH and CH_2_ groups in the glucopyranose ring and acetyl moieties. In native cellulose, broad bands centered at 3289 cm^−1^ and 3350 cm^−1^ are assigned to ν(O–H) stretching of free and hydrogen-bonded hydroxyl groups. These bands are highly sensitive to the degree and geometry of hydrogen bonding, crystalline domain size, and water content. The notable suppression of these bands in cellulose acetate and membrane spectra reflects a collapse of the supramolecular hydrogen-bonding network, an expected outcome of esterification and consistent with increased polymer chain flexibility and reduced crystallinity. This attenuation is also significant from a materials perspective: reduced OH signal intensity implies increased hydrophobicity and reduced moisture sorption, both crucial for biomedical membranes and packaging applications. The similarity of this profile with commercial cellulose acetate validates the chemical equivalence of the cannabis-derived acetate. These vibrational signatures—disappearance of OH-related modes, appearance of CH_3_ and C=O modes, retention of glycosidic bonds are entirely consistent with prior Raman studies of plant-derived cellulose subjected to homogeneous acetylation (Khenblouche et al., 2019). The band patterns align with those reported for cotton linters, microcrystalline cellulose, and hemp-derived acetates, confirming that the transformation pathway used here follows established reaction chemistry. Moreover, the nearly identical spectra between cannabis-derived and commercial membranes confirm that the processed material has achieved a comparable degree of substitution and structural order. This strongly supports the application of *Cannabis sativa* as a viable lignocellulosic feedstock for functionalized biopolymers, while maintaining performance parity with industrial cellulose acetate. For clarity, Table 2 compiles the key vibrational modes observed in both FTIR and Raman spectra, along with their respective functional group assignments.

**TABLE 2:** Summary of characteristic FTIR and Raman vibrational modes and their corresponding functional group assignments in cannabis-derived cellulose acetate.

Wavenumber (cm^−1^)	Technique	Assignment
1736	FTIR	C=O stretching (acetyl ester)
1,372	FTIR	C–H bending of CH_3_ (acetate)
1,220	FTIR	C–O stretching (ester linkage)
1,030	FTIR	C–O–C glycosidic linkage (glucopyranose)
895	FTIR	β-glycosidic ring vibration
1,095	Raman	C–O stretching in cellulose backbone
1,120	Raman	C–C skeletal vibration (cellulose)

### 4.4 Scanning electron microscopy (SEM)

**FIGURE 5:**
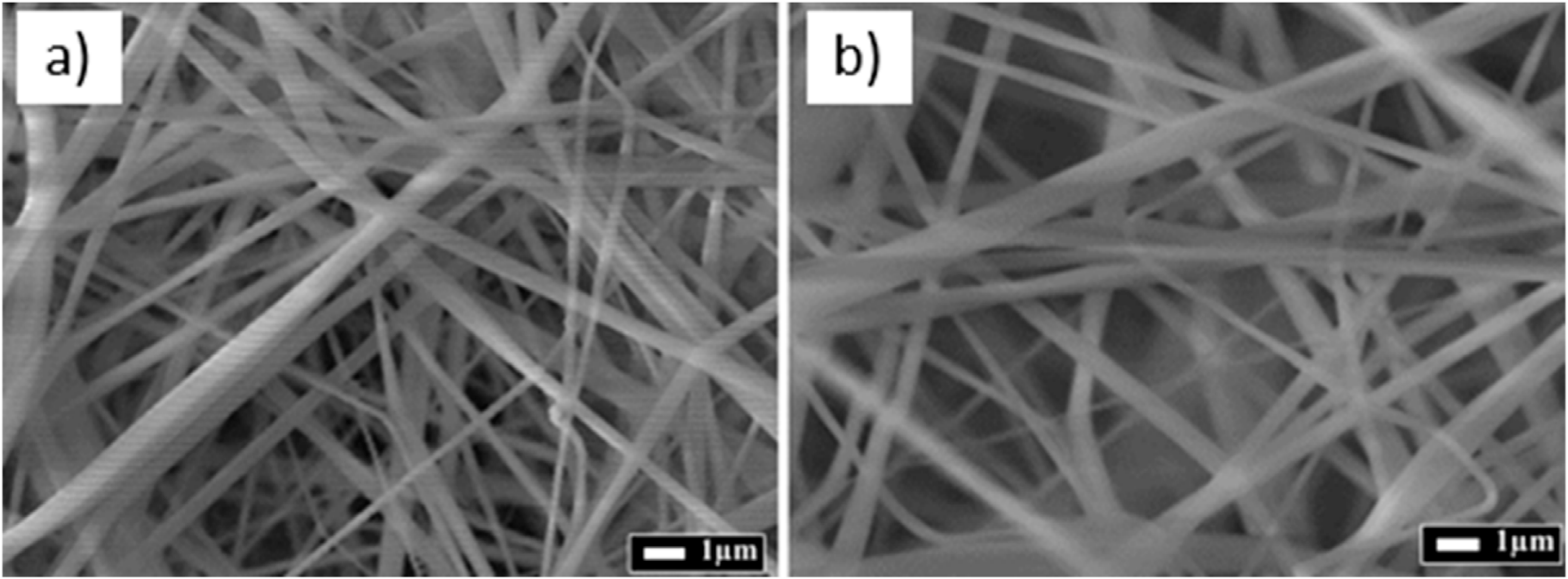
SEM micrographs of commercial cellulose acetate membranes **(a)** and cannabis-modified cellulose acetate membranes **(b)** containing 70% cannabis cellulose acetate and 30% commercial cellulose acetate.

Figure 5 shows the SEM images demonstrate that both membrane types exhibit a continuous, bead-free fibrous morphology typical of stable electrospinning conditions. The fibers are randomly oriented, forming a non-woven, isotropic network with a three-dimensional architecture. This disordered distribution enhances the membrane’s specific surface area and is advantageous for applications requiring surface interaction and fluid permeability, such as therapeutic wound dressings. In Figure 5a the membrane composed solely of commercial CA shows densely packed nanofibers with a relatively uniform diameter and smooth surface topology, indicating homogeneous solution properties and consistent fiber formation dynamics. The dense fiber arrangement suggests a lower pore volume, which may correspond to reduced breathability and fluid absorption characteristics commonly observed in membranes fabricated from fully synthetic, uniform precursors. In contrast, Figure 5b shows a membrane derived from a binary blend of cannabis-based and commercial CA. The fibers retain high morphological fidelity, yet a visibly more open and loosely entangled network is observed. This increased inter-fiber spacing implies enhanced porosity, likely resulting from differences in the physicochemical properties of cannabis-derived CA, such as lower molecular weight, polydispersity, or degree of acetyl substitution all of which influence electrospinnability by modulating viscosity, conductivity, and surface tension of the spinning solution. Importantly, the absence of fused nodes, webbing artifacts, or collapsed structures confirms that the incorporation of bio-derived CA does not compromise fiber integrity or membrane uniformity. At higher magnification, both samples reveal occasional nodular inclusions or spherical features embedded within the fiber matrix. These are hypothesized to originate from microphase separation during jet solidification or localized solvent retention, particularly in cannabis-based formulations, where natural extract residues or minor compositional heterogeneity could alter evaporation kinetics. Despite their presence, these inclusions appear structurally integrated and do not disrupt fiber continuity or network architecture. The SEM analysis confirms that cannabis-derived cellulose acetate is a viable alternative to commercial CA for electrospinning applications. It supports the formation of robust, nanostructured membranes with tunable porosity and uniform morphology, fulfilling key structural criteria for biomedical use. The morphological comparability between the two membrane types validates the successful integration of bio-based CA without adverse effects on fiber architecture, while the enhanced porosity of the cannabis-based membrane may offer added functional benefits in wound management contexts.

### 4.5 UV-Vis spectroscopy

**FIGURE 6:**
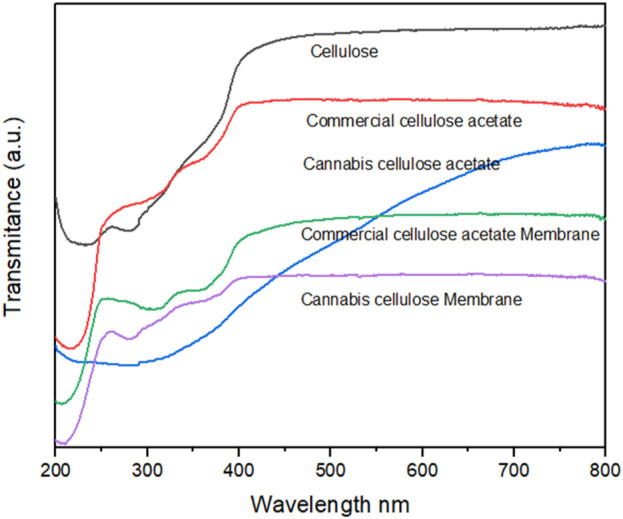
The UV–Vis absorption spectra of pristine cellulose, acetylated cellulose obtained from both commercial and cannabis-derived sources, as well as their respective electrospun membranes.

Figure 6 shows the UV–Vis absorption spectra of pristine cellulose, acetylated cellulose obtained from both commercial and cannabis-derived sources, as well as their respective electrospun membranes. Native cellulose exhibits a steep absorption increase in the far-UV region, with a dominant edge between 200 and 230 nm. This intense band is attributed to π→π* electronic transitions in the oxygen-containing chromophores (C=O, C–O–C, and conjugated hydroxyl groups) that are prevalent in the hydrogen-bonded polysaccharide backbone (Zhang and Jiang, 2023). The sharpness and intensity of this transition are consistent with the strong O–H stretching vibrations (∼3313 cm^−1^) observed in FTIR spectra and the semi-crystalline ordering confirmed by XRD, which shows a characteristic diffraction peak at ∼22.5° 2θ. Following acetylation, both commercial and cannabis-based cellulose acetate samples display broader and red-shifted absorption bands centered between 280 and 300 nm. This bathochromic shift corresponds to the introduction of acetyl groups, which disrupt the hydrogen-bonding network and introduce electron-withdrawing moieties (–COCH_3_), altering the local dielectric environment and reducing the energy gap for electronic transitions. These chemical changes are corroborated by the appearance of strong ester carbonyl stretching bands at 1736 cm^−1^ and methyl deformation vibrations at ∼1,373 cm^−1^ in FTIR spectra, as well as the disappearance of O–H deformation modes in Raman analysis, indicating successful substitution at hydroxyl sites (Mossoba et al., 1996). Additionally, XRD analysis reveals a significant loss of crystalline order in the acetylated samples, with a broad amorphous halo replacing the sharp cellulose reflections, especially in the cannabis-derived variant. The cannabis-based cellulose acetate displays slightly lower absorbance intensity compared to its commercial counterpart. This may reflect a lower degree of substitution, broader molecular weight distribution, or the presence of trace chromogenic impurities (e.g., residual lignin or phenolic compounds), which are more prevalent in biomass-derived materials. These compositional nuances are also reflected in the Raman spectra, which show subtle broadening of C–H and C–O bands, and a more diffuse baseline, indicative of structural heterogeneity. The electrospun membranes of both acetylated systems exhibit further smoothing and attenuation of absorbance, reflecting nanofiber morphology and increased light scattering. SEM analysis confirms the formation of random, interconnected fibrous networks with good fiber uniformity. However, the membrane based on cannabis-derived cellulose acetate shows slightly looser packing and greater surface heterogeneity, which likely contributes to the mild increase in absorbance observed at wavelengths >600 nm a region typically sensitive to scattering and the presence of minor residual chromophores. Importantly, no new spectral features associated with degradation products, e.g., n→π* transitions of carbonyls above 300 nm are observed, suggesting that neither the acetylation process nor electrospinning induces significant oxidative damage or breakdown of the cellulose backbone. The UV–Vis data thus align with the conclusions drawn from vibrational and structural characterization techniques, collectively confirming the successful derivatization of cellulose, preservation of chemical integrity, and optical comparability between cannabis-based and commercial materials.

### 4.6 Obtaining the prototype of the therapeutic dressing

#### 4.6.1 Choosing the best adhesive composition

The influence of cross-linking agent concentration on the cohesion of the adhesive system at both ambient and elevated temperatures is summarized in Table 3. At room temperature (20°C), even low concentrations of the cross-linker were sufficient to reach the upper limit of the test, indicating effective network formation and strong internal cohesion. In contrast, under elevated temperature conditions (70°C), no significant cross-linking effects were observed up to a concentration of 1.0 wt%. However, at concentrations exceeding this threshold, a marked improvement in cohesion was recorded, reaching the experimental maximum. This behavior likely reflects the thermal activation of cross-linking reactions, which promotes greater polymer chain entanglement and enhanced molecular ordering, ultimately resulting in a denser and more structurally coherent adhesive network. These effects are consistent with the trends observed during tack and viscosity testing, where increased cross-linking corresponded with improved film integrity and cohesive performance (Tolia and Kevin Li, 2012; Antosik and Mozelewska, 2022; Licbarski et al., 2022).

**TABLE 3:** Cohesion results of the prepared silicon Q2-7388 composition with various crosslinking agent content.

Crosslinking agent [wt%]	Cohesion [h]	
	20˚C	70˚C
0.0	59.20	0.25
0.5	>72	0.80
1.0	>72	29.20
1.5	>72	>72
2.0	>72	>72
2.5	>72	>72
3.0	>72	>72

The shrinkage behavior of silicone pressure-sensitive adhesives (PSAs) modified with varying concentrations of cross-linking agent is summarized in Table 4. In all cases, dimensional stabilization occurred within approximately 2 days from the initiation of the measurement. Notably, the incorporation of even a small amount of cross-linker (0.5 wt%) led to a substantial reduction in shrinkage, with final values falling below 0.5%, a threshold generally considered acceptable for industrial adhesive applications. Further increases in cross-linker concentration resulted in progressively lower shrinkage values, indicating enhanced dimensional stability. This improvement is likely attributable to the formation of a denser, more ordered polymer network upon cross-linking. The resulting adhesive films exhibit greater structural compactness and reduced free volume, which suppresses relaxation and contraction phenomena typically observed in unmodified formulations. (Antosik et al., 2020; Kang et al., 2021).

**TABLE 4:** Shrinkage of silicone pressure-sensitive adhesives with various crosslinking agent.

Shrinkage [%]												
Crosslinking agent [wt%]	10 min	30 min	1 h	3 h	8 h	24 h	2 Days	3 Days	4 Days	5 Days	6 Days	7 Days
0.0	0.042	0.059	0.071	0.103	0.121	0.134	0.138	0.139	0.140	0.142	0.142	0.142
0.5	0.021	0.056	0.092	0.095	0.099	0.101	0.111	0.109	0.121	0.126	0.129	0.136
1.0	0.042	0.059	0.071	0.079	0.106	0.110	0.123	0.129	0.131	0.134	0.134	0.134
1.5	0.045	0.058	0.071	0.089	0.091	0.096	0.100	0.102	0.106	0.109	0.109	0.109
2.0	0.029	0.045	0.058	0.069	0.078	0.087	0.091	0.092	0.092	0.092	0.092	0.092
2.5	0.037	0.046	0.058	0.076	0.079	0.081	0.083	0.085	0.085	0.085	0.085	0.085
3.0	0.034	0.050	0.056	0.058	0.059	0.062	0.063	0.072	0.077	0.077	0.077	0.077

**FIGURE 7:**
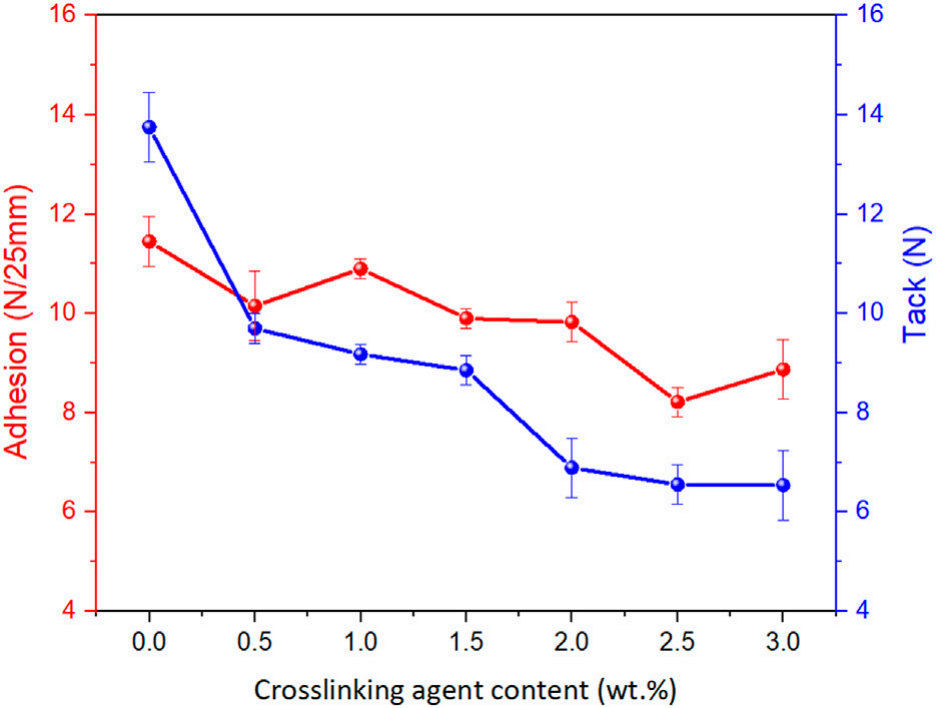
Effect of crosslinking agent content on the peel adhesion and tack of silicone pressure-sensitive adhesive.

Figure 7 presents the quantitative relationship between DClBPO concentration and the adhesive performance of the PSA system, measured in terms of 180° peel adhesion (N/25 mm) and loop tack (N). A consistent downward trend is observed in both parameters with increasing cross-linker content. Even minimal incorporation of the cross-linking agent (0.25–0.5 wt%) results in a substantial reduction in adhesion and tack, indicating that early-stage cross-linking restricts polymer chain mobility and surface wetting behavior. The system containing 1.5 wt% DClBPO achieved a balanced profile, exhibiting adhesion and tack values of 9.9 N/25 mm and 8.55 N, respectively. These values were obtained from triplicate tests (n = 3) and are reported as mean ± SD. The low variability confirms consistent adhesive performance. This concentration appears to represent an inflection point where sufficient network formation occurs to maintain cohesive integrity, while still preserving adequate interfacial interaction with the substrate. At higher concentrations (≥2.0 wt%), further cross-linking leads to reduced tack and adhesion, likely due to excessive rigidity and decreased surface conformability of the adhesive film. These observations suggest that the degree of cross-linking critically governs the viscoelastic balance of the PSA. Increasing the cross-linker content enhances internal cohesion and reduces cold flow, but at the cost of surface bonding strength. This behavior aligns with previous findings (Antosik et al., 2020), where over-crosslinked systems demonstrated diminished interaction with low-energy surfaces. Optimal performance thus requires careful tuning of the cross-linker concentration to balance adhesion, tack, and cohesive strength for targeted biomedical applications.

#### 4.6.2 Research on a simplified skin model simulating the stratum corneum with different humidity

Following a comprehensive evaluation of the adhesive system’s functional performance, including 180° peel adhesion, loop tack, shear cohesion, and dimensional stability under thermal stress a formulation incorporating 1.5 wt% of the cross-linking agent DClBPO (based on polymer dry mass) was identified as the optimal composition. This system exhibited a favorable compromise between adhesive and cohesive properties, minimizing the trade-offs typically observed in pressure-sensitive adhesives (PSAs) upon cross-linking. At this concentration, the polymer network was sufficiently cross-linked to ensure mechanical stability while retaining adequate chain mobility to facilitate substrate interaction and interfacial bonding. To further validate the performance of this formulation under conditions approximating clinical application, adhesion testing was conducted using a synthetic skin surrogate previously developed by the authors. This model, described in a previous investigation by Antosik et al. (2019), consists of a 25:75 blend of carboxymethyl starch (CMS) and carboxymethyl cellulose (CMC). It has been demonstrated to reliably replicate the physicochemical and structural characteristics of the human stratum corneum, particularly in terms of surface energy, hydration sensitivity, and elasticity, which are critical parameters governing the adhesion of biomedical-grade PSAs. Application of the 1.5 wt% DClBPO formulation to this skin-mimicking substrate yielded adhesion and tack values of 7.8 N/25 mm and 6.1 N, respectively. These metrics fall within the operational range of commercially available self-adhesive medical products and confirm the material’s potential for translational use in therapeutic dressings, dermal patches, and related biomedical interfaces. The performance observed further underscores the compatibility of the cannabis-derived cellulose acetate matrix with standard cross-linking strategies and supports its candidacy as a sustainable platform for next-generation bioadhesives.

#### 4.6.3 Prototype

**FIGURE 8:**
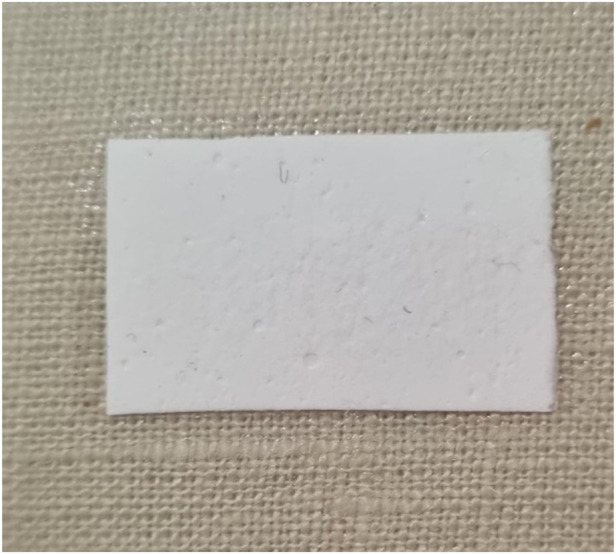
Cannabis cellulose acetate membrane dressing prototype.

A functional prototype was developed by transferring a 45 g/m^2^ PSA onto a nonwoven fabric substrate approximately 50 mm in thickness. A cellulose acetate-based membrane, slightly smaller in area than the fabric, was then laminated onto the adhesive-coated surface in such a way that the peripheral zones of the adhesive remained exposed, enabling self-adhesive functionality at the contact interface. The assembled structure was subsequently covered with a release liner consisting of a commercially available silicone-coated fluorinated polyester film (50 g/m^2^), serving as a protective barrier prior to application (Figure 8). The prototype was fabricated under controlled laboratory conditions and is fully operational in its current form. The use of commercially accessible components, specifically the release film and textile substrate ensures both material availability and reproducibility, while also contributing to the overall mechanical robustness and functional integrity of the patch. This design approach provides a versatile platform for further application in biomedical or transdermal systems.

## 5 Conclusion

This study demonstrates the successful valorization of *Cannabis sativa* biomass into cellulose acetate via an optimized sequence of extraction, purification, and acetylation steps, enabling its subsequent processing into functional electrospun membranes. The resulting membranes exhibited a well-defined, three-dimensional nanofibrous architecture with high porosity and uniform fiber distribution morphological features that are highly desirable for filtration and biomedical applications. The electrospinning process, optimized at a polymer concentration of 17 wt%, consistently produced uniform membranes with randomly oriented nanofibers. SEM analysis confirmed that both commercial and cannabis-derived cellulose acetate membranes formed interconnected networks with increased surface area, suitable for applications requiring controlled permeability and interfacial contact. Structural and chemical characterization using FTIR, Raman, and XRD verified the successful acetylation of cellulose, preserving the glucopyranose backbone while introducing acetyl functionalities. The modified structure retained key spectral signatures consistent with acetylated polysaccharides, while a discernible reduction in crystallinity was observed evidenced by broadened diffraction peaks and characteristic spectral shifts. This partial amorphization is advantageous for biomedical applications, as it improves flexibility, diffusivity, and potential drug incorporation capacity. The membranes retained physicochemical features compatible with biomedical use, particularly in wound dressings and drug delivery systems. Although *in vitro* biological testing was not conducted in this study, the biomedical relevance of the membranes is supported by the chemical identity and structural similarity of the cannabis-derived CA to commercial cellulose acetate, a clinically approved and extensively validated material. Spectroscopic and morphological analyses confirmed chemical purity and structural uniformity. Nonetheless, biological performance including cytocompatibility (MTT assay), microbial inhibition, and cell-material interactions remains to be evaluated and is planned in future work to complete the translational validation. UV–Vis spectroscopy further corroborated these findings, showing that cannabis-derived cellulose acetate membranes possess optical properties closely resembling those of their commercial counterparts. Minor variations in absorbance were attributed to differences in molecular weight distribution or residual plant-derived compounds, without compromising overall optical performance. Although quantitative data on swelling behavior and pore size distribution were not collected in this study, SEM images confirmed a highly porous, interconnected nanofiber network typical of electrospun materials. Such architecture is widely recognized to promote moisture exchange, cellular infiltration, and oxygen permeability key factors in wound healing. In parallel, mechanical integrity was indirectly assessed through FINAT-standard peel, tack, and shear testing, demonstrating robust performance under simulated clinical use. The absence of tensile strength and swelling assays is acknowledged as a limitation, and these measurements are planned for future validation stages. *Cannabis sativa* biomass contains bioactive phytochemicals such as cannabinoids and flavonoids, the alkaline–oxidative purification process effectively removes these compounds to ensure chemical purity. Spectroscopic analyses (UV–Vis, FTIR) confirmed the absence of residual chromophores, indicating complete removal of phytoconstituents. Consequently, the wound healing potential of the membrane is attributed to its nanofibrous architecture and physicochemical properties, rather than to any retained therapeutic molecules. Functional evaluation of the adhesive formulations revealed a systematic decrease in adhesion and tack with increasing cross-linker content. The composition containing 1.5 wt% DClBPO was identified as optimal, achieving a favorable balance between adhesion (9.9 N/25 mm) and tack (8.55 N). This formulation was selected for prototype development based on its mechanical cohesion and practical applicability. This contributes to circular bioeconomy goals by transforming agricultural residues into clinically relevant devices. For benchmarking purposes, the observed adhesive performance was compared with typical ranges reported for medical-grade pressure-sensitive adhesives under 180° peel testing conditions. The cannabis-derived cellulose acetate system exhibited a peel strength of 9.9 ± 0.4 N/25 mm and tack of 8.55 ± 0.3 N, positioning it within the upper performance range for such materials. These results suggest that the developed formulation meets the adhesive performance requirements for clinical use, while offering a biobased and sustainable alternative. The final prototype, fabricated using commercially available textile substrates and release liners under laboratory-scale conditions, exhibited mechanical integrity and functional versatility. Adhesion tests conducted on synthetic substrates mimicking human skin confirmed the prototype’s suitability for self-adhesive medical applications, such as transdermal patches or wound-contact layers. Taken together, this work presents the first full validation of cannabis-derived cellulose acetate as a processable, biocompatible, and functionally versatile material for advanced medical dressing systems. Collectively, these findings support the use of *Cannabis sativa* as a viable and sustainable raw material for the development of high-performance cellulose acetate membranes. The study demonstrates not only the chemical and morphological comparability of cannabis-derived materials to commercial analogs but also their potential in next-generation biomedical and filtration technologies.

## 6 Limitations and future work

**FIGURE 9:**
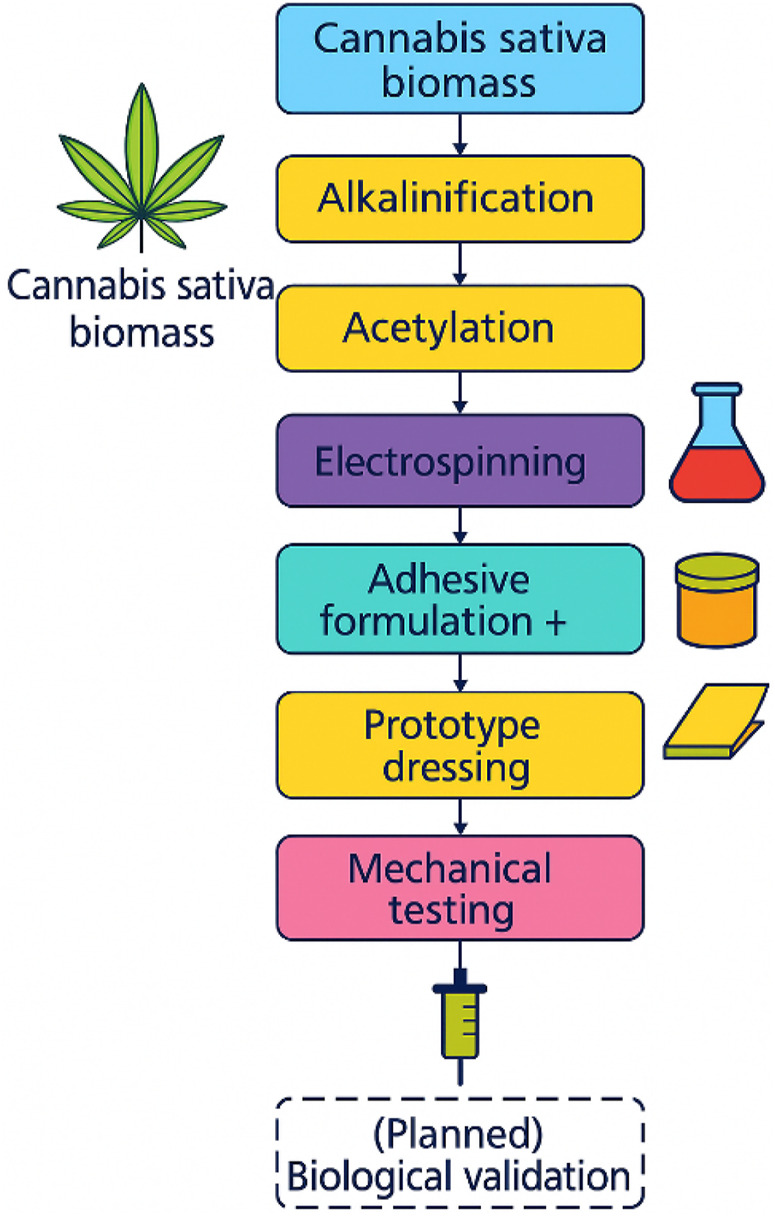
Visual summary of the fabrication and testing workflow for cellulose acetate membrane-based adhesive dressings derived from *Cannabis sativa* biomass.

While this study demonstrates the feasibility of synthesizing high-purity cellulose acetate membranes from *Cannabis sativa* and integrating them into adhesive wound dressing prototypes, several limitations must be acknowledged. No biological assays were conducted to assess cytocompatibility, hemocompatibility, or antimicrobial properties. Likewise, *in vivo* or *ex vivo* performance data were not collected. Future research will therefore include standard cytotoxicity testing, microbial inhibition assays, and wound healing evaluation in animal models, aligned with international standards for biocompatibility assessment (e.g., ISO 10993 series). Additionally, translational steps toward clinical validation will be prioritized, including testing on skin-mimicking models, long-term stability studies under physiological conditions, and assessments of sterilization compatibility. These steps are essential to prepare the developed membranes for preclinical evaluation and medical device classification. From a sustainability perspective, subsequent efforts will aim to enhance the environmental profile of the process by incorporating green solvents, optimizing energy consumption, and implementing closed-loop solvent recovery systems. Future work will explore mechanical separation techniques like sieving or flotation to enhance scalability and reproducibility. Upscaling under good manufacturing practice (GMP) constraints and a life cycle assessment (LCA)-based evaluation will also be pursued to ensure both ecological feasibility and clinical relevance. A schematic overview of the complete fabrication and evaluation process is provided in Figure 9, highlighting the workflow from biomass sourcing to dressing prototype testing.

## Data Availability

The original contributions presented in the study are included in the article/supplementary material, further inquiries can be directed to the corresponding author.
